# Unique gating strategy identifier based on the Prime Population System and Gödel Numbers

**DOI:** 10.3389/fimmu.2025.1481412

**Published:** 2025-02-12

**Authors:** Antonio Cosma

**Affiliations:** National Cytometry Platform, Translational Medicine Operations Hub, Luxembourg Institute of Health, Esch-sur-Alzette, Luxembourg

**Keywords:** cytometry, prime numbers, database, ontology, gating strategy

## Abstract

Gating is a fundamental and essential process in cytometry data analysis since it defines cell types of interest. Currently, there is no universally accepted method for representing and sharing gating strategies among software, publications, and repositories. I propose using the Prime Population system combined with a modified version of Gödel Numbers to identify any gating strategy uniquely. The Prime Population System is first used to identify gates on bivariate plots; successively, Gödel Numbers are used to set the sequence of the hierarchical gating strategy. The process results are unique identifiers for any existing and future gating strategy. The unique identifiers hold since according to the fundamental theorem of arithmetic, every natural number other than one either is a prime or is a product of prime numbers, and every nonprime number can be factored into a product of prime in only one way. This method represents a further step towards the arithmetization of cytometry metadata.

## Introduction

Gating is an essential step in flow cytometry data analysis. It involves defining regions or gates in the data corresponding to specific cell populations based on marker expression on univariate or bivariate plots. Hierarchical gating consists of creating a series of gates, each nested within the previous one, to identify and quantify specific cell populations of interest. Despite the proliferation of automated methods to define cell populations, such as clustering and dimensionality reduction, manual gating remains the gold standard for characterizing a cell population (1). Moreover, hierarchical gating is how populations are defined for fluorescence-based single-cell sorting.

The gating strategy procedure is described in manuscripts using figures inserted in the main text or supplementary materials. As a matter of example, axis are named without any convention (e.g.: PD1, PD-1, CD279, PD1-155Gd, CD279-PE …) and gates regions are labeled with free text without a strict relationship to the axis used to define them. Thus far, there is no standard way to represent a gating strategy and exchange this information among software, publications, and repositories.

I previously proposed a standard to name markers and cell types based on prime numbers and the fundamental theorem of arithmetic (2). An ordered list of markers is associated with the list of prime numbers according to a one-to-one relationship. The choice of the marker catalog is crucial to define the application domain, and in my previous manuscript, I anticipated using the UniProt for cytometry (https://www.uniprot.org/). The product of the prime numbers associated with the markers creates a unique composite number uniquely defining a cell type. Inversely, the composite number can be factorized to obtain the original primes and the markers associated with a specific cell type. I named the principle Prime Population System (PPS). The PPS ensure a unique correspondence between the markers and a unique composite number associated to the cell type. The unique correspondence hold true since the fundamental theorem of arithmetic state that “every natural number other than 1 either is a prime or is a product of prime numbers, and every nonprime number can be factored into a product of prime in only one way” (3). Therefore the PPS ensures a unique tag for every cell type without concertation among stakeholders and without arbitrary assignment of names or codes. Importantly, the PPS tag is precisely linked to the underlying biology via the one-to-one correspondence between markers and prime numbers.

I propose implementing a Unique Gating Identifier (UGI) based on the PPS and a modified version of Gödel Numbers. Kurt Gödel invented the Gödel Numbers to demonstrate the first incompleteness theorem (4). The discovery and the proof of the incompleteness theorem represent one of the most significant contributions to logic since Aristotle. In the framework of the proof, Gödel used a combination of products of powers of prime numbers to define uniquely any element of the arithmetic system (5).

To describe any element of a gating strategy uniquely, I modified the Gödel numbers, integrating the concept of markers and populations as described in the PPS. The UGI expands the capability of the PPS to set up a unique correspondence between any given gating strategy and a number derived by a product of primes.

## Methods

**Figure 1:**
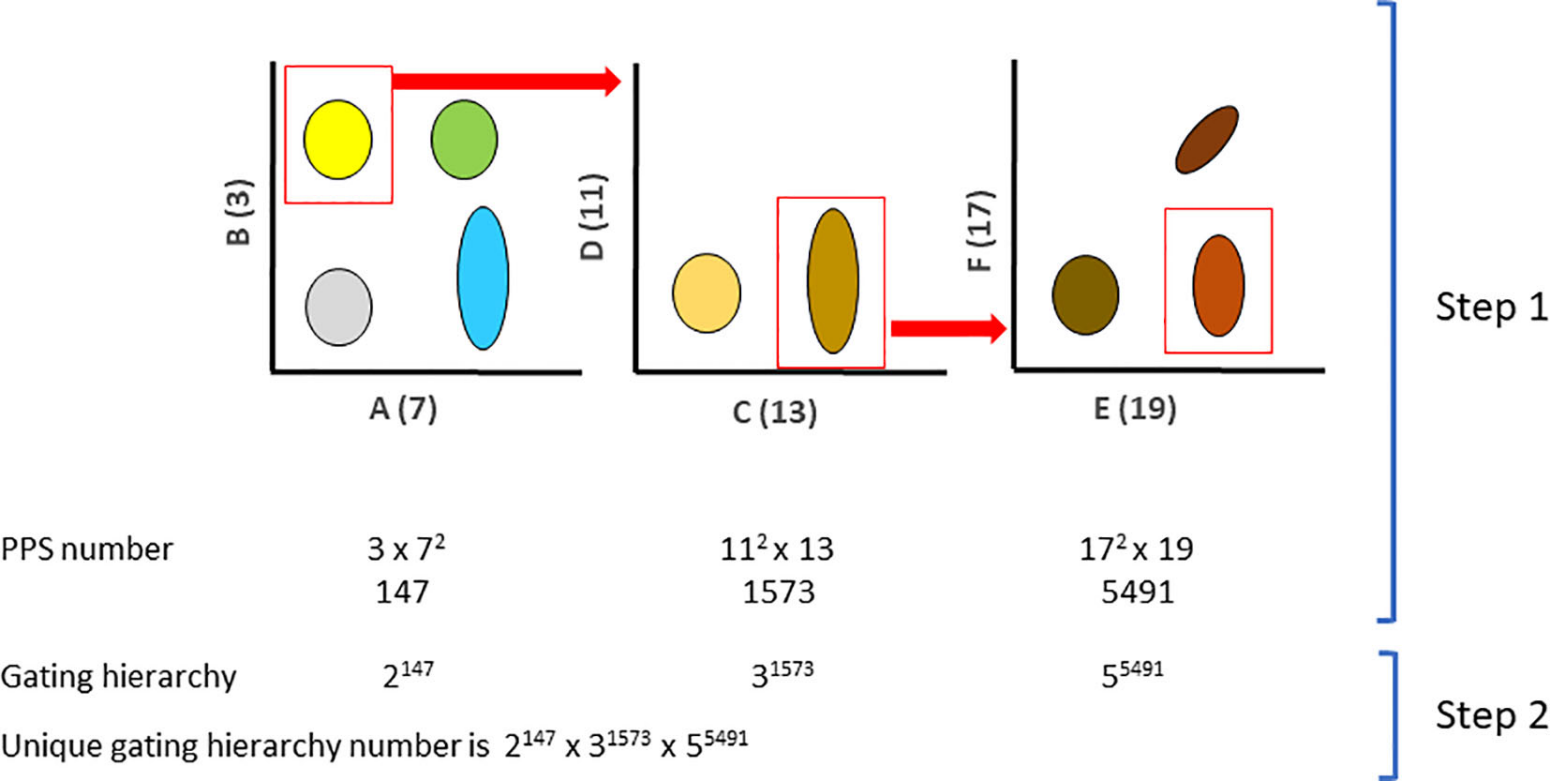
Schematic of the UGI principle. (Step 1) Markers A, B, C, D, F, and G were associated with the prime numbers 3, 7, 11, 13, 17 and 19 following the PPS rules. A PPS number is generated for every sequential gate. (Step 2) A sequential prime number is assigned to each hierarchical gate, and it is raised to the respective PPS.

The UGI method proceeds in two steps (**Figure 1**). The first step consists of associating prime numbers to the UniProt list to have a functional PPS as previously described (2). The list of prime numbers and the associated list of proteins is available at the following link (https://public.tableau.com/app/profile/fanny2212/viz/PrimeNumbers-UniProt/PrimeNumbers-UniProtassociation). The association uses the UniProt list downloaded on January 18^th^, 2023. Prime numbers two and five were skipped since they might be used in future implementations of the arithmetization since they have the properties to generate easily identifiable products, i.e., even numbers and numbers ending with zero or five. Each gate on a bivariate plot is considered a cell type and uniquely described by a PPS number according to the fundamental theorem of arithmetics. The second step consists of associating each gate with a consecutive prime number to reflect the sequence of the hierarchical gating. Finally, each sequential prime number is raised to the PPS number describing the respective hierarchical gate. The UGI is the product of the successive prime numbers raised to the PPS number. The resulting product is uniquely associated with a specific gating strategy. Since we perform products of prime numbers, the resulting composite number will be unique for each existing and future gating strategy. Of note, the fundamental theorem of arithmetic holds when the same prime is used repeatedly in the product, equivalent to raising a prime to the n power. Therefore, assigning a unique number to each gating strategy is possible. Moreover, since the PPS is able to describe intermediate expression by raising the prime number to the power of three (2), more complex gates can be described at each step of the hierarchical gating strategy. Per extension, the PPS may simply describe a more complex gate definition by dividing each dimension along the axis and assigning consecutive powers to each region.

Practically, in any experiment, one UGI will correspond to each final cell type identified at the end of the hierarchical gating strategy. The UGI will represent a unique tag to identify which gating strategy was used to identify every cell type.

## Discussion

**Figure 2:**
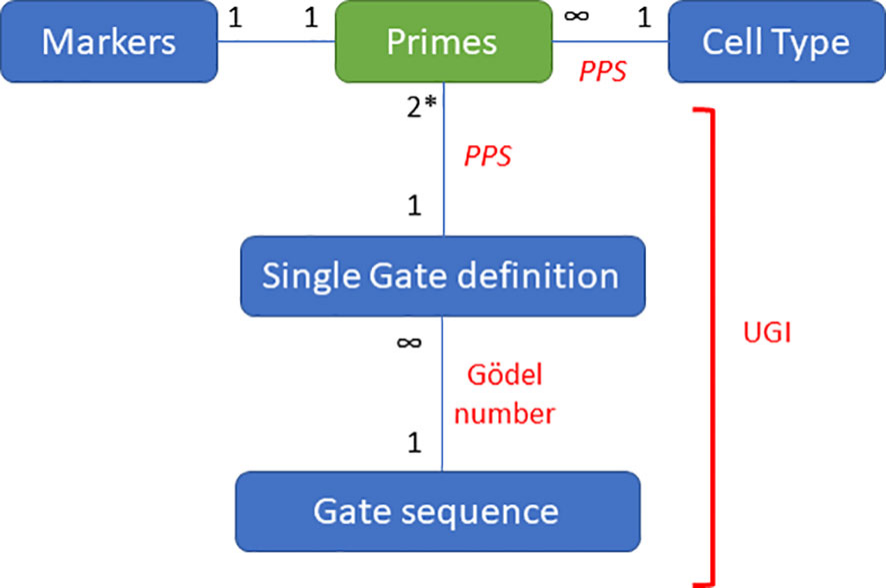
Relationship among the objects of arithmetization, the rules governing the system, and prime numbers. The objects of arithmetization are shown in blue with an edge describing the relationships with the list of primes. The rules governing the system are depicted in red. *Two axes are considered in this example; nevertheless, the system holds for univariate plots and even gates based on multiple markers such as Boolean gates.

The UGI number assures a unique identifier for any existing hierarchical gating strategy based on the Prime Population System (PPS) and Gödel Numbers. Similar to the PPS that identifies cell types, the UGI is unique for every existing gating strategy that has been drawn in the past and will be drawn in the future. Considering the present manuscript and the previous description of the PPS (2), **Figure 2** schematizes the relationships among the objects of arithmetization, the system’s rules, and the prime numbers. Prime numbers and the fundamental theorem of arithmetic represents the basis of the system. The markers, cell types, gates, and sequence of gates are the objects of the arithmetization whereas the PPS, the modified Gödel Numbers and the UGI are the rules governing the relationship among the objects.

The UGI numbers can be used in databases, manuscripts, and cytometry software to exchange information about gating strategies. Since the UGI is linked to the identified cell type via the PPS, the capacity of the two systems allows the integration of information generated in different experimental settings. It is essential to note that, leveraging the PPS, the UGI number connects the gating strategy with the biology of the identified cell type since cell type and gating strategy are coded in the same system (**Figure 2**).

Gating-ML 2.0 (6) is a standard supported by the International Society for the Advancement of Cytometry (ISAC) that unambiguously describes gates and data transformation to exchange this information among different software tools. It handles information about type and sequence of conventional gates, compensation, scale transformation, and custom metadata. However, the standard does not propose a unique way to identify a gating strategy in relation to the biological markers and, therefore, it is limited to the experimental domain. Indeed, there is no convention to name the markers inside Gating-ML. However, UGI numbers can be added inside the metadata section of Gating-ML at the gate level and render the standard able to build connections among different experiments. One of the advantages of the arithmetization presented here is the possibility of searching for markers and populations and comparing gating strategies among multiple experiments. For instance, it is straightforward to identify Th1, Th2, and Th17 CD4+ T cell subsets, but the gating to define the parent memory CD4+ T cell population can be different according to panel complexity (7). Indeed, the definition of memory CD4+ T cells may include the exclusion of CD56+ cells, gamma-delta T-cells, or T regulatory cells. This information is rarely searchable, standardized, and often available only as supplementary figures. The UGI will associate a unique code with every gate, and the order of the primes will clearly define the hierarchy.

Automated gating methods have been developed to reduce user-to-user variability and increase the speed of analysis (8). In the cluster analysis framework, when hundreds of clusters are generated in every experiment, it is impossible to name them via a meaningful biological string. The PPS system will be useful to label with a unique tag every cluster. The information can then be compared to any other data set using the PPS, and when the dataset is also analyzed by a conventional hierarchical gating, the gating strategy can be retrieved via the UGI.

A limitation of the present standard is that cleaning gates, such as the one used to identify doublets in flow or in mass cytometry (8), are not considered. Since cleaning gates are not based on biological markers, they cannot be included in the system and must be represented in a separate format. At the moment this part of the gating strategy more technical and not linked to the biology is out of the scope of the UGI.

The idea behind the arithmetization of cytometry starting from prime numbers is to build a precise framework to code for cell types and gating strategies and, in the future, for other characteristics of the cells, such as the expression profiles, cell-to-cell interactions, and spatial relationships. Every characteristic will be precisely linked to the original prime and successively to the one-to-one relationship with the UniProt database. The PPS and UGI recast the relationship among markers, cell types, and gating strategy in terms of the fundamental concept of number theory. Thus building a base for future implementations.

The system I describe has vocation to work in the foreground of software, database, and repositories. This manuscript aims to propose a theoretical system to the cytometry community; any implementation will be useful only when integrated with a cytometry software complete with all the conventional functions.

Existing software tools can easily manage numbers generated by the PPS (2). By instance, Python 3 int data type, supports arbitrary precision, which means it can represent integers of any size, limited only by the available memory on the system. Nevertheless, the product of sequential primes raised to the PPS will generate numbers that will be difficult to manage. UGI may be handled, for instance, as vectors of bases and powers. I imagine every software company willing to implement the arithmetization will choose the best solution for their environment. This is primarily engineering and out of the scope of the present manuscript. The unique identifier concept holds independently from the way everyone will process the UGI expressions. For example, the number 5491 can be written as a product of primes: 172 x 19, hexadecimal: 1573, or in scientific notation, 5*1000 + 4*100 + 9*10 + 1*1. The underlying meaning will remain the same: a cell type negative for marker F and positive for marker E. It remains a unique identifier. The PPS and UGI represent a common language to share information and keep a meaningful link to biology independently of how they are represented inside a software package. For instance, number theory holds independently of the numerals used to describe it.

The main barrier to adopting PPS and UGI is that they need to be implemented as a standard by the overall community to be helpful. The adoption by a single laboratory or software company will not be as effective as the collective effort of the entire community. The addition of UGI to the PPS is already a step forward to increase the attractiveness of the system, and future development may convince stakeholders that this is a valid solution to standardize cell biology.

Finally PPS and UGI code for cell types and gating strategies using the same language, and this represents an enormous advantage in terms of interoperability and information sharing among software. Since the proposed arithmetization provides an unambiguous link to biology, the system may be expanded to encode additional cell characteristics in a mathematical language.

## Data Availability

Publicly available datasets were analyzed in this study. This data can be found here: https://public.tableau.com/app/profile/fanny2212/viz/PrimeNumbers-UniProt/PrimeNumbers-UniProtassociation.
